# Extra-Cellular But Extra-Ordinarily Important for Cells: Apoplastic Reactive Oxygen Species Metabolism

**DOI:** 10.3389/fpls.2017.01353

**Published:** 2017-08-22

**Authors:** Anna Podgórska, Maria Burian, Bożena Szal

**Affiliations:** Institute of Experimental Plant Biology and Biotechnology, Faculty of Biology, University of Warsaw Warsaw, Poland

**Keywords:** antioxidants, apoplastic reactive oxygen species, biotic and abiotic stress, detoxification, respiratory burst enzymes

## Abstract

Reactive oxygen species (ROS), by their very nature, are highly reactive, and it is no surprise that they can cause damage to organic molecules. In cells, ROS are produced as byproducts of many metabolic reactions, but plants are prepared for this ROS output. Even though extracellular ROS generation constitutes only a minor part of a cell’s total ROS level, this fraction is of extraordinary importance. In an active apoplastic ROS burst, it is mainly the respiratory burst oxidases and peroxidases that are engaged, and defects of these enzymes can affect plant development and stress responses. It must be highlighted that there are also other less well-known enzymatic or non-enzymatic ROS sources. There is a need for ROS detoxification in the apoplast, and almost all cellular antioxidants are present in this space, but the activity of antioxidant enzymes and the concentration of low-mass antioxidants is very low. The low antioxidant efficiency in the apoplast allows ROS to accumulate easily, which is a condition for ROS signaling. Therefore, the apoplastic ROS/antioxidant homeostasis is actively engaged in the reception and reaction to many biotic and abiotic stresses.

## Introduction

The term reactive oxygen species (ROS) usually encompasses singlet oxygen (^1^O_2_), superoxide ion ($O_{2}^{•–}$), hydrogen peroxide (H_2_O_2_), and hydroxyl radical (OH⋅); however, other molecules, such as organic hydroperoxides (ROOH), are also included in this group (Simon et al., 2000). ROS can be generated in all cellular compartments through non-enzymatic mechanisms, such as electron flow in electron transport chains (ETC), or as byproducts of enzymatic reactions (Desikan et al., 2005). ROS are reactive and cause damage to macromolecules (proteins, lipids, and nucleic acids) affecting cellular functioning, but more recent evidence indicates that ROS play important roles in signaling and cell adaptation to stress. Mechanisms of intracellular ROS production and ROS detoxification have been extensively reviewed (Halliwell, 2006; Das and Roychoudhury, 2014); however, apoplastic ROS (apROS) metabolism has received less attention.

Only a small quantity of ROS is localized within the apoplast, apROS have an important role in plant development and plant responses to various stress conditions. We elucidate how apROS are engaged in signal transduction from extracellular spaces to the cell interior and may directly eliminate invading pathogens. Moreover, apROS metabolism regulates the plant cell division rate and cell elongation, and consequently whole plant growth (Barceló and Laura, 2009). It was shown that one of the components regulating cell proliferation is apoplast-localized low-mass antioxidants (Kato and Esaka, 1999; Potters et al., 2000). Growth by elongation is limited by the extension of cell walls in what is the result of two processes in which ROS are engaged: loosening of load-bearing bonds in the wall matrix and stiffening through the insertion of stabilizing cross-links between load-bearing polymers (Chen and Schopfer, 1999; Pedreira et al., 2004; Kärkönen and Kuchitsu, 2015). The presence of ROS in apoplastic spaces has mainly been demonstrated using microscopic methods (e.g., deposits of cerium peroxides observed in TEM or probes that became fluorescent when oxidized by ROS), but precise concentrations of apROS were also estimated in some plant species. The concentration of H_2_O_2_ in apoplastic fluid was about 10–25 pmol⋅g^-1^ FW under control conditions and increased several fold under stress conditions (Hernández et al., 2001; Diaz-Vivancos et al., 2006). The question remains how the apoplastic respiratory burst does not become toxic for plants. Our aim in this update is to provide a concise overview of current knowledge about long known ROS producing mechanisms and also propose less prominent apoplastic ROS sources. Furthermore we discuss data regarding antioxidant capacity in the apoplast.

## Apoplastic ROS Formation

### NADPH Oxidases

**FIGURE 1:**
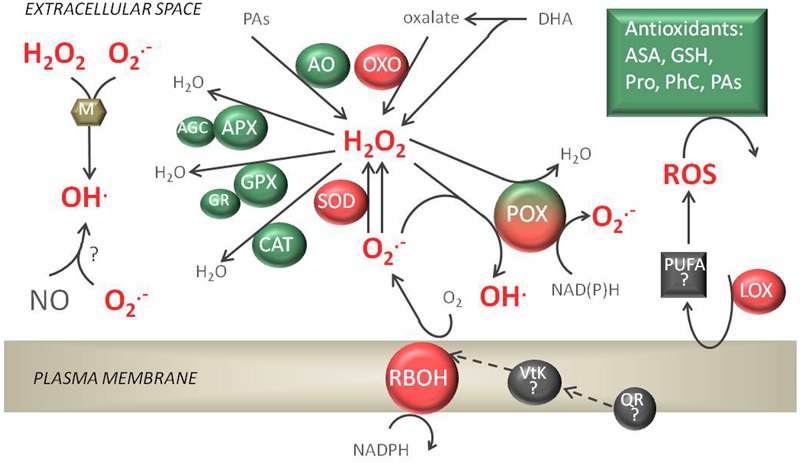
Apoplastic reactive oxygen species (ROS) production and detoxification reactions. The respiratory burst oxidase homolog (RBOH) enzymes, in cooperation with superoxide dismutases (SODs), peroxidases (POXs), amine oxidases (AOs), and oxalate oxidases (OXOs), are responsible for H_2_O_2_ production. Cell wall POXs forming a triple loop can also catalyze the formation of $O_{2}^{•–}$ and HO⋅ or H_2_O_2_ detoxification. Among enzymatic antioxidants present, ascorbate peroxidase (APX), performing a whole ascorbate glutathione cycle (AGC), glutathione peroxidase (GPX), and catalase (CAT) can be detected in the apoplast. The non-enzymatic antioxidant reduced ascorbate (AsA) and glutathione (GSH), proline (Pro), phenolic compounds (PhC), and polyamines (PAs) are soluble in apoplastic fluids. Non-enzymatic sources of ROS are also possible in the presence of metal cofactors (M) or nitric oxide (NO). Lipoxygenase (LOX) activity can produce Lipid peroxidation products (polyunsaturated fatty acids, PUFA) which can possibly generate ROS.

Plant NADPH oxidases, termed respiratory burst oxidase homologs (RBOHs), are located in the plasma membrane and generate apoplastic $O_{2}^{•–}$ using cytosolic NADPH as an electron donor (**Figure 1**). Plant NADPH oxidases are composed of six transmembrane domains and cytosolic FAD- and NADPH-binding domains (Kadota et al., 2015). The cytoplasmic N-terminal region of plant NADPH oxidases contains two highly conserved EF-hand motifs that are responsible for regulating RBOH activity; however, conformational changes mediated by Ca^2+^ binding occur in only one EF-hand (Oda et al., 2010). Calcium-dependent activation of RBOH activity is also regulated by protein phosphorylation (Kimura et al., 2012), RBOH-lipids (Zhang Y. et al., 2009), and RBOH-specific protein interactions (Kawarazaki et al., 2013; Kaur et al., 2014). The activity of plant RBOHs has to be tightly regulated to diverse stimuli. However, in opposite to animal systems, only a few proteins that interact to the N-terminal extension of plant RBOHs and mediate positive or negative regulation of its activity has been found (Sumimoto, 2008). Recently, a low temperature-inducible protein AtSRC2 was found to activate AtRBOHD (Kawarazaki et al., 2013). It is known that conformational changes of phosphorylated RBOHs protein expose them to interaction with Rac GTPases (Wong et al., 2007). Rac GTPases participates in a wide variety of signaling events in plants (Nagawa et al., 2010). Several Rac homologs have been shown to activate plant RBOHs (Kawasaki et al., 1999; Wong et al., 2007). On the other hand Rac5 protein in *N.icotiana tabacum* down-regulated *NtRbohD* (Morel et al., 2004). Among other type of protein interactions the binding of calcineurin B-like protein (CBL)-interacting protein kinase 26 (CIPK26) to AtRBOHF was shown. CIPK26-RBOH association negatively modulated activity of RBOH (Kimura et al., 2013). A role for extracellular ATP in RBOH activity regulation was also suggested (Song et al., 2006). RBOHs belong to a multigene family: 10 *RBOH*s were identified in *Arabidopsis*, 9 in *Oryza sativa*, and 2 in *N. benthamiana* (Marino et al., 2012; Kaur et al., 2014). Individual *RBOH*s differ in their expression pattern across plant tissues and organs, suggesting that their function is diversified. At*RBOH* expression was roughly divided by Sagi and Fluhr (2006) into three categories according to the places of their occurrence: expression in whole plant (At*RBOHD* and *F*), in roots (AtRBOH *A*–*G, I*), and in pollen (At*RBOHH* and *J*), but in other studies it was shown that, e.g., At*RBOHB* is expressed in germinating seeds (Müller et al., 2009).

### Peroxidases

Peroxidases (POXs) can be differentiated into class I POXs, which are intracellular, and class III POXs (E.C. 1.11.1.7) which are secreted to the vacuole or exported to the extracellular space. POXs are heme-containing enzymes involved in ROS production (in hydroxylic and oxidative cycles) and elimination (peroxidative cycle, discussed later) (**Figure 1**). The activity of POX in an oxidative cycle (using NADH as a substrate) or peroxidative cycle (using phenolics as a substrate) was measured in EWF (extracellular washing fluids) from different zones of maize root and leaf (Maksimović et al., 2008). In the oxidative cycle POXs reduce O_2_ to $O_{2}^{•–}$ or H_2_O_2_ using apoplastic reductants, and in the hydroxylic cycle POXs catalyze the reaction in which OH⋅ is produced from H_2_O_2_ and $O_{2}^{•–}$ (Chen and Schopfer, 1999). In the *Arabidopsis* genome 73 POX isoforms were identified (Tognolli et al., 2002) – among these only 6 were predicted not to be secreted to the apoplast (Valério et al., 2004). Using multidimensional protein identification technology, it was possible to identify proteins tightly bound to the cell wall, and several POXs in the cell wall proteome (POX1, 2, 12, 30, 31, 57, 64) were revealed (Bayer et al., 2006). In a novel approach, plasmalemma-bound POX isoforms were identified that seemed to have N-terminal transmembrane domains (Mika et al., 2008). POX genes are mainly expressed in roots, but they have also been detected in other plant organs (Tognolli et al., 2002). A crucial element in the production of ROS by POXs appears to be a transient alkalinization of the apoplast (Bolwell et al., 1995). The regulation of 7 POX genes by KUA1 [a transcription factor (TF) upregulated during expansion growth of leaves] alludes to the particular role of these enzymes in developing processes (Schmidt et al., 2016 and references therein). It was also shown that in *Arabidopsis* POXs are responsible for about 50% of the H_2_O_2_ produced during the oxidative burst in pathogen defense (O’Brien et al., 2012a). Additionally, apROS generated by POXs can activate the transcription of RBOHs, which allows further accumulation of apROS in response to stress (O’Brien et al., 2012a).

### Oxalate Oxidases

Oxalate oxidases (OXOs; EC 1.2.3.4) belong to the germin-like protein (GLP) family. GLPs have been reported to possess different enzyme activities, such as superoxide dismutase and polyphenol oxidase, and some of them possess OXO activity (Li et al., 2016). The best characterized are OXO in Poaceae (Berna and Bernier, 1997), but OXO activity has also been detected in other plants, e.g., in *Silene vulgaris* (Bringezu et al., 1999). OXOs are Mn-containing oligomeric proteins that catalyze the oxidative breakdown of oxalate into CO_2_ and H_2_O_2_. All OXOs are characterized by their secondary structure. They are formed as a β-sheet containing a short internal α-helix and a C-terminal α-helical region (Membré et al., 1997). Thirty-seven GLP genes were identified in the *Arabidopsis* genome (Li et al., 2016). The presence of GLPs in *Arabidopsis* was associated with the extracellular matrix; nevertheless, none of the *Arabidopsis* GLPs have OXO activity (Membré et al., 2000). H_2_O_2_ generated by OXOs is involved in the defense response in certain plant–pathogen reactions (Hu et al., 2003).

### Amine Oxidases

Amine oxidases (AOs) include the copper-containing AOs (CuAOs; EC 1.4.3.6) and the flavin-containing polyamine oxidases (PAOs; EC 1.5.3.11). CuAOs catalyze oxidative deamination of aliphatic diamines such as putrescine (Put) and cadaverine (Cad), and less efficiently spermine (Spm) and spermidine (Spd), at the primary amino groups. PAOs are responsible for the oxidation of Spm, Spd, and their acetylated derivatives at the secondary amino group. In both reactions H_2_O_2_ is generated. *Arabidopsis* contain 10 genes encoding CuAOs, of which only eight encode for putative functional proteins. Among them AtCuAOβ (At1g62810, previously named AtCuAO1) and AtCuAOγ1 (At4g14940, previously ATAO1) are extracellular proteins (Planas-Portell et al., 2013; Tavladoraki et al., 2016). In the *Arabidopsis* genome, there are five genes encoding PAOs (Tavladoraki et al., 2006), but according to the conducted studies none of these AtPAOs are located in the apoplast (Tavladoraki et al., 2016 and references therein); apoplastic localization of PAOs was shown in barley and maize (Cervelli et al., 2001; Cona et al., 2006).

### Other apROS Sources

In addition to the apROS sources listed above, other mechanisms of apROS production occur in plant tissues (**Figure 1**). The plant plasmalemma-localized phylloquinone (vitamin K1) can transport electrons from the cytosolic NAD(P)H-dependent quinone reductase (QR), and it was hypothesized that this vitamin donates electrons to RBOHs leading to apROS production (Bridge et al., 2000). The non-enzymatic stepwise degradation of ascorbate may also generate ROS (Green and Fry, 2005). Additionally, lipoxygenase (LOX) causes hydroperoxidation of polyunsaturated fatty acids (PUFA) making it an active source of ROS (Das and Roychoudhury, 2014). OH⋅ can be generated in a reaction between nitric oxide (NO) and $O_{2}^{•–}$, initially generating peroxynitrite (ONOO^-^). NO is produced in apoplasts from nitrate by plasmalemma-bound nitrate reductase (PM-NR) and nitrite:NO reductase (NI-NOR) (Cohen et al., 2014). Hydroxyl radicals can also be generated in the apoplast in a metal-catalyzed Fenton/Haber-Weiss reaction.

### ROS Scavenging in the Apoplast

Reactive oxygen specie scavenging pathways are responsible for maintaining a low steady-state baseline of ROS. Apoplasts, similar to intracellular cell compartments, are characterized by a specific set of antioxidants (**Table 1**) that can detoxify all types of ROS produced there (Mittler et al., 2004).

**Table 1:** Annotation of extracellular localization of antioxidant enzymes.

Enzyme	Agi	TAIR	SUBA4	ePLANT
**Superoxide Dismutases**				
FSD1	At4g25100	Plasma membrane	Extracellular: SLP-Local; MS Plasma membrane: MS	Extracellular: 12 Plasma membrane: 50
FSD2	At5g51100	No extracellular annotation	Plasma membrane: MS	Plasma membrane : 10
CSD1	At1g08830	No extracellular annotation	Extracellular: EpiLoc; PredSL; MS	Extracellular: 14
CSD2	At2g28190	apoplast	Extracellular: SubLoc; MS	Extracellular: 2
CSD3	At5g18100	No extracellular annotation	Extracellular: SubLoc; MS	Extracellular: 12
MSD1	At3g10920	No extracellular annotation	Extracellular: MS	No extracellular annotation
MSD2	At3g56350	No extracellular annotation	extracellular: Predotar; PredSL; PProwler; iPSORT; SLP-Local; WoLF PSORT; Target P; YLoc; MS	Extracellular: 16
**Ascorbate Peroxidases**				
APX1	At1g07890	No extracellular annotation	Extracellular: MS	Plasma membrane: 30
APX2	At3g09640	No extracellular annotation	Extracellular: PredSL	Extracellular: 2
APX3	At4g35000	No extracellular annotation	Extracellular: MS	Plasma membrane: 20
APX6	At4g32320	Extracellular region	No extracellular annotation	No extracellular annotation
**Monodehydroascorbate Reductases**				
MDHAR6	At1g63940	No extracellular annotation	Extracellular: MS	Plasma membrane: 10
MDHAR3	At3g09940	No extracellular annotation	Extracellular: MS Plasma membrane: MS	No extracellular annotation
MDHAR4	At3g27820	Integral component of membrane	Extracellular: Predotar; PredSL; PProwler; SLPFA; SLP-Local; BaCelLo; iPSORT Plasma membrane: MS	Extracellular: 14; Plasma membrane: 10
MDHAR1	At3g52880	Apoplast, plasma membrane	Extracellular: MS Plasma membrane: MS	Plasma membrane: 50
MDHAR2	At5g03630	No extracellular annotation	Extracellular: MS Plasma membrane: MS	No extracellular annotation
**Dehydroascorbate Reductases**				
DHAR2	At1g75270	Plasma membrane	Extracellular: AdaBoost; SLPFA Plasma membrane: MS	Extracellular: 4; Plasma membrane: 20
DHAR4	At1g19550	No extracellular annotation	Extracellular: SLPFA	Extracellular: 2
DHAR1	At1g19570	Apoplast, plasma membrane	Extracellular: AdaBoost; SLPFA; MS Plasma membrane: MS	Extracellular:4; Plasma membrane: 30
**Glutathione Reductases**				
GR1	At3g24170	No extracellular annotation	Extracellular: MS	No extracellular annotation
GR2	At3g54660	No extracellular annotation	Extracellular: MS	No extracellular annotation
**Catalases**				
CAT1	At1g20630	Cell wall	Extracellular: SLPFA; SubLoc Plasma membrane: MS	Extracellular: 4; Plasma membrane: 10
CAT2	At4g35090	No extracellular annotation	Extracellular: SLPFA; SubLoc; MS Plasma membrane: MS	Extracellular: 4 Plasma membrane: 20
CAT3	At1g20620	Apoplast, cell wall, plasma membrane,	Extracellular: SLPFA Plasma membrane: MS	Extracellular: 2 Plasma membrane: 80
**Glutathione Peroxidases**				
GPX1	At2g25080	No extracellular annotation	Extracellular: MS	No extracellular annotation
GPX2	At2g31570	Plasma membrane	Extracellular: PredSL Plasma membrane: MS	Extracellular: 2 Plasma membrane: 10
GPX3	At2g43350	No extracellular annotation	Extracellular: PProwler Plasma membrane: AdaBoost	Extracellular: 2 Plasma membrane: 2
GPX4	At2g48150	No extracellular annotation	Extracellular: PredSL; SLPFA Plasma membrane: MS	Extracellular: 4 Plasma membrane: 10
GPX5	At3g63080	Plasma membrane	Extracellular: PredSL Plasma membrane: MS	Extracellular: 2 Plasma membrane: 60
GPX8	At1g63460	No extracellular annotation	Extracellular: PredSL; SLPFA; MS	Extracellular: 4

*The *Arabidopsis* Information Resource (TAIR) proposes the intracellular localization of enzymes on the basis of literature. The SUBcellular localisation database for *Arabidopsis* proteins (SUBA4) suggests the localization of enzymes on the basis of different bioinformatics software or mass spectrometry (MS). The Bio-Analytic Resource for Plant Biology (ePLANT) proposes a score for the localization within a plant cell on a heat scale.*

### Superoxide Dismutases

Superoxide dismutases (SODs, EC 1.15.1.1) constitute the first enzymatic defense against ROS; these enzymes convert $O_{2}^{•–}$ into H_2_O_2_, a less harmful reactant. SODs are classified, according to the metal cofactor used by the enzyme, as iron (Fe-), manganese (Mn-), or copper-zinc (CuZn-) dependent (Kliebenstein et al., 1998; Alscher et al., 2002). A specific set of SOD isozymes is present in all cellular compartments. In *Arabidopsis* CuZnSOD2 (At2g28190) was identified in EWF by isotope labeling (Bindschedler et al., 2008). Moreover, in a proteomic approach, one of the FeSOD isoforms was found to be an integral protein of the plasma membrane represented by the *Arabidopsis* gene At5g25100 (Marmagne et al., 2004, 2007).

Superoxide dismutases activity in the apoplast was estimated at only 0.13–2.5% of symplastic SOD activity; it was also shown that CuZnSOD is the most active apoplastic isoform (Vanacker et al., 1998a,b; Hernández et al., 2001; Kukavica et al., 2005). Moreover, the distribution of CuZnSOD proteins was analyzed in spinach mesophyll cells by immunogold electron microscopy, and revealed that up to 44% of gold particles were located in the apoplast compared to other cell compartments (Ogawa et al., 1996). Most CuZnSODs are localized in proximity to the plasma membrane, but proteins were also found in the secondary thickening cell wall (Ogawa et al., 1996). Moreover, Ogawa et al. (1997) proposed that CuZnSOD proteins are closely attached to RBOH. Therefore, CuZnSOD may be the major source of H_2_O_2_ in the apoplastic space, after dismutation of $O_{2}^{•–}$ produced by RBOHs.

### Catalases

Catalases (CATs, EC 1.11.1.6) can directly catalyze a dismutase reaction of two molecules of H_2_O_2_ to water and O_2_. Catalases are unique among antioxidants since they can decompose ROS without the need for any reductant. The catalase gene family in *Arabidopsis* is represented by three isozymes, CAT1-3 (Frugoli et al., 1996). Even though the major functional site of CATs are peroxisomes, the isozymes CAT1 (At1g20630) (Bayer et al., 2006) and CAT3 (At1g20620) (O’Brien et al., 2012a) were also found to be connected with the cell wall. Salguero and Böttger (1995) expected CAT activity to be tightly connected to ROS production by RBOH or POX in the apoplastic space. Catalases have been detected in xylem cell walls of sunflower cotyledons by immunogold electron and immunofluorescence microscopy (Eising et al., 1990) and in isolated cell wall fractions from horseradish (Elstner and Heupel, 1976). It was shown that CAT activity in the apoplast represented only 0.2–2% of total foliar enzyme activity in barley and oat (Vanacker et al., 1998a,b). Interestingly, in some experiments it was shown that the CAT activity in the apoplastic EWF was about three times higher than that in the soluble symplastic fraction (Parra-Lobato et al., 2009).

### Peroxidases

In the peroxidative cycle POXs catalyze the reduction of H_2_O_2_ by taking electrons from various molecules, mainly phenolic compounds or auxin. Since POXs utilize H_2_O_2_ as a substrate, their catalytic activity reduces H_2_O_2_ levels; therefore, these enzymes can also be considered as antioxidants (Chen and Schopfer, 1999). Apart from the cytosolic fraction, which showed the highest POX activity in tobacco suspension cultures and the liverwort *Dumortiera hirsuta*, the extracellular space was associated with almost half as much peroxidase activity (Kawano et al., 1998; Li et al., 2010). Among analyzed cell wall fractions, Li et al. (2010) found the highest enzyme activity for loosely bound peroxidases (21% of total activity). However, the activity of POXs bound by hydrophobic interactions, strong electrostatic forces, or covalent linkages should not be ignored. In sunflower seedlings activity of peroxidase ionically bound to the cell wall was higher than that of covalently bound or free apoplastic isozymes (Parra-Lobato et al., 2009). It is possible that during stress conditions, or an apoplastic oxidative burst, some peroxidase isoforms can be released from the cell wall to the apoplast to protect the plants. Since there is such a multiplicity of POX isoforms, it was speculated that these enzymes might show a functional specialization (Cosio and Dunand, 2009). On the basis reported data the proposed functions of individual POXs were listed in the review by Cosio and Dunand (2009) or by Shigeto and Tsutsumi (2016). The regulation of POX activity should be discussed at least at three levels: at the genes expression level and control of protein secretion to apoplast, post-translational modifications, and substrate specificity *in planta*. Promoter sequences of *Arabidopsis* POXs are highly diversified, suggesting specific transcriptional regulation (Francoz et al., 2015). The different pattern of POX expression and/or secretion results in presence or absence of specific isoforms during diverse physiological processes or their localization in different organs. Post-translational regulation of POX activity include: the influence of apoplastic pH – different isoforms of POX (anionic, cationic, or neutral) may be activated or repressed in that way (Minibayeva et al., 2015; Mangano et al., 2016); protein modification in processes glycosylation and/or phosphorylation (Van Huystee et al., 2004; Mika et al., 2008) and regulation of POX activity by calcium (Carpin et al., 2001). Diversity and availability of POX substrates have also a high impact on POX function *in vivo*. *In vitro* POXs have broad substrate specificities, although it is unclear which of these compounds function as substrates *in planta* (Cosio and Dunand, 2009).

## Enzymes of the Ascorbate-Glutathione Cycle

**FIGURE 2:**
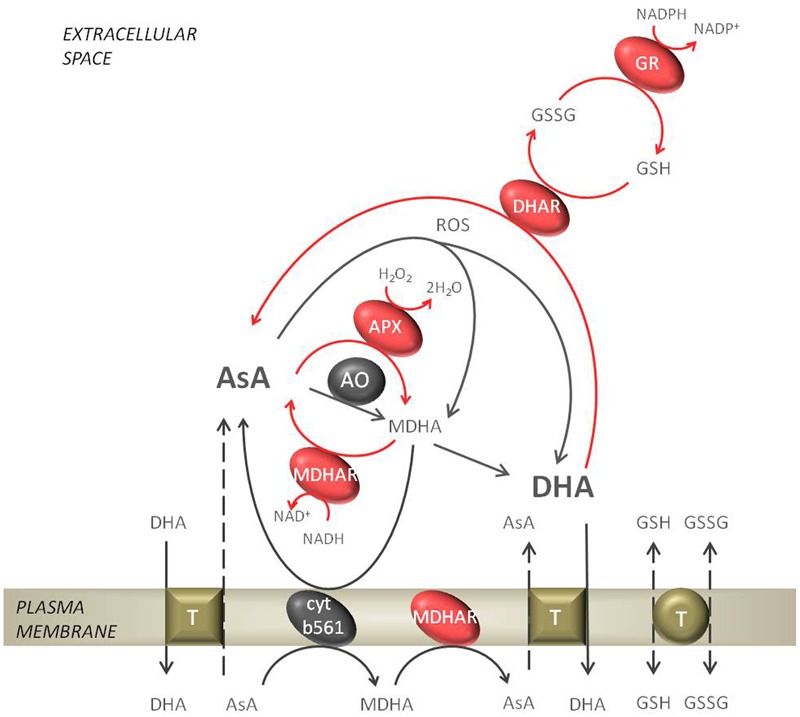
Ascorbate metabolism in the apoplastic space. The reduced form of ascorbate (AsA) gets oxidized by ascorbate oxidase (AO) or APX to form monodehydroascorbate (MDHA). The reverse reaction, where MDHA gets reduced to AsA can be performed by the plasmalemma bound cytochrome b561 or monodehydroascorbate reductase (MDHAR). In an non-enzymatic mechanism MDHA disproportionate spontaneously to dehydroascorbate (DHA). Further, DHA can be reduced to AsA by dehydroascorbate reductase (DHAR), which is dependent on reduced glutathione (GSH). In the regeneration of GSH glutathione reductase (GR) is engaged. The exchange of GSH/GSSG and AsA/DHA from the apoplast to the cytosol by putative transporters is indicated. The network of reactions of the ascorbate-glutathione cycle are indicated with red arrows.

The combined action of ascorbate peroxidase (APX, EC1.11.1.11), dehydroascorbate reductase (DHAR, EC 1.8.5.1), monodehydroascorbate reductase (MDHAR, EC 1.6.5.4), and glutathione reductase (GR, EC 1.6.4.2) can decompose H_2_O_2_ using ascorbate (AsA) and glutathione (GSH) as electron donors, via a pathway called the ascorbate-glutathione cycle (AGC, **Figure 2**). In the AGC the reduction of H_2_O_2_ is ultimately linked to NAD(P)H oxidation. All AGC enzymes were found in the apoplasts of barley, oat, and pea leaves, but their activity was estimated at only 0.2–2.8% of symplastic activity (Vanacker et al., 1998a,b; Hernández et al., 2001). However, in the apoplasts of other plants not all AGC enzymes were found to be active; therefore, it is also possible that the whole cycle is not operating in the apoplast, but only certain enzymatic reactions (De Pinto and De Gara, 2004).

The first enzyme of the AGC, APX, is a class I peroxidase that uses AsA as an electron donor to scavenge H_2_O_2_ to water. In the *Arabidopsis* genome eight types of APX were found, but only APX1 (At1g07890), which is considered a cytosolic enzyme, might also be secreted to the apoplast. APX1 was identified in several reports of extracted plasmalemma or cell wall proteins (Marmagne et al., 2007; O’Brien et al., 2012a). APX showed the highest activity of all AGC enzymes (Diaz-Vivancos et al., 2006), and in roots of sunflower seedlings apoplastic APX activity was even higher than that detected in the soluble symplastic fraction (Parra-Lobato et al., 2009). APX has an even higher affinity for H_2_O_2_ (μM range) than CATs and class III POXs (mM range).

The regeneration of AsA from its oxidized state is necessary for APX activity. MDHAR is a key enzyme for the recovery of AsA within plant cells, using NAD(P)H directly to reduce monodehydroascorbate (MDHA). The MDHAR multigene family is encoded by the *MDHAR1-6 Arabidopsis* genes that are targeted to most cellular compartments. Among these proteins MDHAR1 (At3g52880) seems to be localized in organelles and also in the apoplastic space (Bindschedler et al., 2008). Additionally, a plasma membrane-associated MDHAR was found in spinach and identified by NMR (Bérczi and Møller, 1998). However, the *in vivo* localization of MDHAR was shown to be on the cytosolic side of the plasma membrane.

Dehydroascorbate reductase catalyzes the reduction of dehydroascorbate (DHA) to AsA, but the enzyme activity requires GSH as an electron donor. DHAR is thought to be responsible for the regulation of the symplastic and apoplastic AsA redox state. In *Arabidopsis* DHAR is encoded by *DHAR1-3* genes. DHAR1 (At1g19570) and DHAR2 (At1g75270) were found to also be associated with the plasma membrane (Marmagne et al., 2007). The reduction of DHA by GR simultaneously results in GSH oxidation.

Glutathione reductase catalyzes the NADPH-dependent reduction of glutathione disulfide (GSSG) to the sulfhydryl form of GSH. GR regenerates GSH and thereby fuels the AGC; therefore, GR is thought to be the rate-limiting step of the cycle. In the apoplast of barley and oat this enzyme has the lowest activity among AGC enzymes in EWF (Vanacker et al., 1998a,b). There seem to be only two genes representing GR in *Arabidopsis*, GR1 and GR2; however, to date no extracellular isoform has been identified in *Arabidopsis*.

The *in vivo* activity of GR and MDHAR in the apoplast remains a matter of debate since their substrate, NAD(P)H, seems to be present at very low concentrations in this space. In fact, there is no evidence in the available literature of reduced pyridine nucleotides being extracted from apoplastic fluids. However, the oxidized form of pyridine nucleotides (NAD^+^) which usually constitutes the major amount of nucleotides, was detected in EWF of needles of Norway spruce at a level up to 1.4 nmol⋅g^-1^ DW (Otter and Polle, 1997). O’Brien et al. (2012b) proposed a cycle in which NADH can be produced, involving the action of apoplastic malate dehydrogenase (MDH) or lactate dehydrogenase (LDH) using NAD^+^ as a substrate.

## Other Extracellular Enzymatic Antioxidants

Glutathione peroxidases (GPXs, EC 1.11.1.9) are important for the detoxification of peroxides, such as H_2_O_2_, alkyl hydroperoxides, and peroxynitrite, using GSH as a substrate. In *Arabidopsis* eight isoforms of GPXs can be found (GPX1-8); of these GPX2 (At2g31570), GPX5 (At3g63080), and GPX6 (At4g11600) were found in the plasma membrane (Marmagne et al., 2007). In sunflower seedlings GPX activity in the apoplast did not account for even 10% of symplastic enzyme activity (Parra-Lobato et al., 2009). Some peroxidases favor thioredoxin (TRX, EC 1.8.1.9) over GSH as a substrate in their regeneration system. Moreover, in the antioxidative system, 2 further thiol-dependent peroxidases are important: peroxiredoxins (PRXs) and glutaredoxins (GRXs). PRXs are reduced by TRX while GRX reduction depends on reduced GSH. These thiol-based POXs cannot only eliminate hydroxyperoxides, but GPXs, PRXs, and GRXs can revert protein disulfides to dithiols and can therefore protect protein thiols from oxidation. Some of these PRXs and GRXs might be secreted to the extracellular space, but detailed characterization of these enzymes is lacking (Meyer et al., 2008).

### Ascorbate

The reduced form of ascorbate (AsA) can scavenge ROS as a consequence of being oxidized to form MDHA and DHA (Smirnoff, 1996). In the apoplastic space of most plants the ascorbate content was estimated to reach up to 10% of the total cellular pool and is in the micromolar range (Vanacker et al., 1998a; Pignocchi and Foyer, 2003). The low apoplastic ascorbate content might be connected to AsA degradation in this space. In contrast to cytosolic ascorbate, in the apoplast the pool is generally more oxidized; the quantity of ascorbate in the oxidized state reaches 75–99% (Takahama and Oniki, 1992; Vanacker et al., 1998a,b; Hernández et al., 2001; De Pinto and De Gara, 2004). Ascorbate can be oxidized by ROS, but the apoplastic enzyme ascorbate oxidase also catalyzes the oxidation reaction of AsA to MDHA (Pignocchi and Foyer, 2003). The resulting MDHA spontaneously disproportionates to AsA and DHA, accelerated by the acidic apoplastic pH (Parsons and Fry, 2012).

The possibility of efficient reduction of extracellular DHA and MDHA is still a matter of debate. It was proposed that the transmembrane protein cytochrome b561 can transfer electrons from cytosolic AsA to extracellular acceptors to reduce MDHA to AsA on the apoplastic side (Horemans et al., 1994). Also plasmalemma-bound MDHAR plays a role in the regeneration of AsA from MDHA (Bérczi and Møller, 1998). Another possibility to reduce apoplastic DHA is via transfer to the symplast and exchange for AsA (**Figure 2**). Horemans et al. (1998) proposed a model of ascorbate transport at the plasma membrane of higher plant cells involving specific transporters. Maurino et al. (2006) identified in *Arabidopsis* putative plasmalemma ascorbate transporters sharing similarity with known nucleobase-ascorbate transporters (NATs) from other species. DHA appears to be the preferred form of transport from the apoplast to the cytosol through high-affinity carriers or facilitated diffusion (Smirnoff, 1996). Infiltration of DHA into tobacco cell cultures or leaves of silver birch proved that active transport of ascorbic acid across the plasma membrane is necessary to achieve reduction of DHA occurring inside the cytosol (Potters et al., 2000; Kollist et al., 2001). However, the transport system seems to be too slow to maintain a reduced ascorbate pool in the apoplastic space during high rates of ROS production (Horemans et al., 2000). Therefore, the quest for discerning ascorbate transport systems across the plasma membrane needs definitely further research.

### Glutathione

Glutathione is an abundant tripeptide (γ-glutamylcysteinglycine) in plant tissues (reviewed by Noctor et al., 2012). The reduced form of glutathione (GSH) can scavenge all ROS and is itself oxidized to glutathione disulphide (GSSG). Using biochemical methods, glutathione was localized and measured in all cellular compartments, including the apoplast (Vanacker et al., 1998a,b; Hernández et al., 2001); unfortunately the gold particle immunocytochemical method used to label glutathione in the apoplasts of *Arabidopsis* leaves was below the level of detection (Zechmann et al., 2008). In most plant species, around 1–10% of the total glutathione pool is present in the apoplastic space. In some plant species, such as barley and pea, the apoplastic pool was estimated to be in the micromolar range (Vanacker et al., 1998a,b; Hernández et al., 2001). The low glutathione content in the apoplast might be due to localization of GSSG degradation in this space. Hydrolysis of glutathione is carried out by *g*-glutamyltransferase/transpeptidase (GGT), which is probably bound to the cell wall of plants (Storozhenko et al., 2002). GGT promotes the transfer of glutamate mainly from GSSG to other dipeptides, but also GSH and GS-conjugates can be utilized in a lower extent. In *Arabidopsis* and other plant species, GGT1 and GGT2 are associated with the cell wall (Ohkama-Ohtsu et al., 2007). Glutathione transport could be accomplished by the oligopeptide transporter (OPT) family, which can transfer GSH, GSSG, and GS-conjugates, among other peptides, across the plasmalemma (Zhang et al., 2004). During the import of glutathione both GSH and GSSG compete for the same carrier transport systems (Zhang et al., 2004). However, the efficiency of glutathione export to the apoplast remains a matter of debate and no putative transporters were identified up to date (Foyer et al., 2001; Noctor and Foyer, 2016). The apoplastic glutathione pool is generally more oxidized than the cytosolic glutathione pool. Approximately 45–55% of the apoplastic glutathione pool was found to be in the oxidized state (Vanacker et al., 1998a,b; Hernández et al., 2001). Glutathione, similar to ascorbate affects cell cycle progression in plant cells. Exogenous application of GSH stimulates cells undergoing mitosis and might, therefore, promote cell division during hair tip growth of the *Arabidopsis* root or in tobacco cell suspensions (Sánchez-Fernández et al., 1997; Potters et al., 2004). However, an increased GSSG level does not have a negative effect on cell proliferation, as was reported for DHA (De Pinto et al., 1999).

## Other Antioxidants

In the apoplast other low-mass antioxidants are also present, such as phenolics and polyamines, which might be important for ROS detoxification. Phenolics are a heterogeneous category of molecules containing a phenol group. Almost 10000 compounds can be assigned to this group, including diverse secondary metabolites. Many of these phenolics (flavonoids, tannins, hydroxycinnamic acid, and lignin) possess antioxidant properties. As electron donors, phenolic compounds can quench ROS because they are easily oxidized, and the resulting phenoxyl radicals are less reactive than oxygen radicals (Kagan and Tyurina, 1998). Polyphenols were tested *in vitro* and found to be the most efficient low-mass antioxidants in plant cells (more effective than AsA or tocopherols) but were not as effective as SOD (Taubert et al., 2003). Moreover, phenolics are also able to scavenge lipid radicals and prevent the propagation of lipid peroxidation. The major form in which phenolics can be detected is bound to the cell wall (Wallace and Fry, 1994; Hutzler et al., 1998). However, free phenolic compounds were also detected in EWF (Maksimović et al., 2008). Beyond that it was found that the composition of phenolics in the apoplast can change in response to different elicitors (Baker et al., 2015). Phenolics can be released into the apoplastic space either through vesicle-mediated transport or specific membrane transporters, including GST-flavonoid complexes. However, the ROS scavenging capacity of phenolic compounds in the apoplastic space was estimated not to be efficient enough, probably because of the low concentration (Baker and Mock, 2004). It is known that phenols can activate POX activity, since they serve as their substrate. The reaction products – phenoxyl radicals – have to be further detoxified via reduction by AsA. Therefore, phenols that are oxidized by POXs can form a ROS-scavenging phenolic/ASA/POX system, in which the action of MDHAR is also necessary to reduce the resulting MDHA (Takahama and Oniki, 1992). Among phenolic compounds, POXs can utilize flavonoids as their substrate. Flavonoids are one of the major classes of phenolics, with the ability to serve as direct electron donors; therefore, they can scavenge all kinds of ROS (Bors et al., 1990). In plant cells flavonoids can be transported via transporters or vesicles to the apoplast and accumulate mainly within cell walls (Zhao, 2016). Another category of phenolics includes acidic derivatives such as hydroxycinnamic acid and hydroxybenzoic acid. Hydroxycinnamic acid is of great interest because of its antioxidant features (hydrogen- or electron-donating ability). Hydroxycinnamic acids (such as ferulic, caffeic, sinapic, and p-coumaric acids) are structural and functional constituents of plant cell walls (Wallace and Fry, 1994). The oxidation of derivatives of hydroxycinnamic acid by peroxidases is one of the features that make them important in the apoplast.

Polyamines were assumed to be protective compounds (Bouchereau et al., 1999). The most abundant plant polyamines include Putrescine, Spermidine, and Spermine. However, the catabolism of polyamines occurs in the apoplast; therefore, their content can be very low or even at undetectable levels. The concentration of different polyamines in EWFs of tobacco and barley was determined to be around 1–10 μM (Marina et al., 2008; Sobieszczuk-Nowicka et al., 2016). Due to their cationic nature at physiological pH, polyamines are able to interact with negatively charged macromolecules in a reversible manner, which suggests that they may be free radical scavengers (Ha et al., 1998; Das and Misra, 2004). However, the oxidative degradation of polyamines in the apoplast by AOs can produce significant quantities of H_2_O_2_; therefore, their role as antioxidants is questionable (Rea et al., 2004). The direct beneficial effect of polyamines for plant development or defense responses is still a matter of research.

The amino acid proline (Pro) is not only an important osmolyte in plant cells, but also an important scavenger of ROS. Pro utilizes OH⋅ and ^1^O_2_, and can inhibit the damage caused by the chain reaction of lipid peroxidation (Matysik et al., 2002). The reactivity of Pro with H_2_O_2_ and $O_{2}^{•–}$ seems very low; therefore, the role of Pro as an efficient cellular antioxidant has been questioned (Kaul et al., 2008). The content of Pro in apoplasts was estimated at around 1–20 μM in the EWF of bean and sugarcane (Tejera et al., 2006; Sobahan et al., 2009).

Some hormones belonging to the indole family show high reactivity with ROS, allowing the indoleamine to function as an electron donor and serve as a trap for ROS chain reactions. Even though these molecules are mostly known as mammalian hormones, some indole derivatives, such as tryptophan, melatonin, and auxins, are present in plants. The most prominent mammalian indole is melatonin, and its role as an antioxidant in plants has also been recognized (Arnao and Hernández-Ruiz, 2006). To date, indole derivatives have shown various different biological effects, other than acting as antioxidants. In plants auxins are the most studied among indole derivatives that regulate cell growth (Leyser, 2010). In recent years, their ROS decomposing activity became more apparent, and some indoles are even more active than GSH (Cano et al., 2003). The most valuable feature of indoles is their small size. Moreover, they are both water and lipid soluble; hence these molecules can easily penetrate the plasma membrane, but their antioxidant potential in the apoplast has to be further elucidated.

The plasma membrane has a lipophobic center that most antioxidants cannot enter; therefore, lipid-soluble vitamins are important for membrane protection. Two of the different forms of vitamin E are tocopherols and tocotrienols. In contrast to other low-mass antioxidants, tocopherols are hydrophobic and lipid-soluble and because of their proximity to membranes tocopherols can therefore protect lipids and other membrane components from lipid peroxidation (Munné-Bosch and Alegre, 2002). Among the α, β, γ, and δ forms of tocopherols, α-tocopherol is most abundant in leaves and is also the most active form (Kaiser et al., 1990). Tocopherols can directly quench ROS, especially ^1^O_2_ which is irreversibly oxidized. Regeneration of the resulting tocopheroxyl radical to its reduced state is performed by AsA or GSH (Fryer, 1992). Moreover, tocopherols can scavenge lipid peroxyl radicals considerably faster, before they are able to attack the target lipid substrate. The concentration of α-tocopherol in membranes is relatively low (less than 2 mol/mol phospholipid), and since no transport mechanism of these molecules from plastids (where they are synthesized) has been detected to date, its content in the plasma membrane is questionable. Nevertheless, it was shown that the lack of tocopherols in mutants resulted in cell wall defects such as callose deposition (Maeda et al., 2006).

The electrons from vitamin K1 might also be used to reduce oxidized lipids in plasma membranes, terminating lipid peroxidation chain reactions. A possible electron acceptor might be cytochrome b561, simultaneously causing AsA regeneration.

The main method to isolate the apoplastic fluids is the infiltration of leaves and extraction of EWF. In previous studies where the concentration of ascorbate and glutathione or antioxidant enzyme activity was determined, it is important to be aware of the possibility of contamination of this EWF fraction with cytosolic components. It is not possible for these low mass antioxidants to freely diffuse through the membranes to the apoplast, but small disruption of the plasma membrane cannot be excluded during the experimental procedure extracting EWF. The presence of glucose 6-phosphate dehydrogenase (G6PDH) activity can be used as a marker for cytoplasmic contamination of EWF. In all the described studies less than 2% of total extractable foliar G6PDH activity was found in the EWF.

Large scale proteomics of EWF revealed that most proteins are stress-related or cell wall-modifying enzymes (Rodríguez-Celma et al., 2016); therefore, there is no doubt over the apoplast’s function in defense. Nevertheless, a complex antioxidant network has evolved in the apoplastic spaces of plants, although the overall antioxidant defense in this space is generally low compared to that in the intracellular space. The low ROS detoxification capacity in the apoplastic space provides a fast overflow mechanism that allows ROS to accumulate. The aim of this respiratory burst may be the precondition for ROS signaling.

## ROS Signaling Connecting the Apoplast with the Nucleus

**FIGURE 3:**
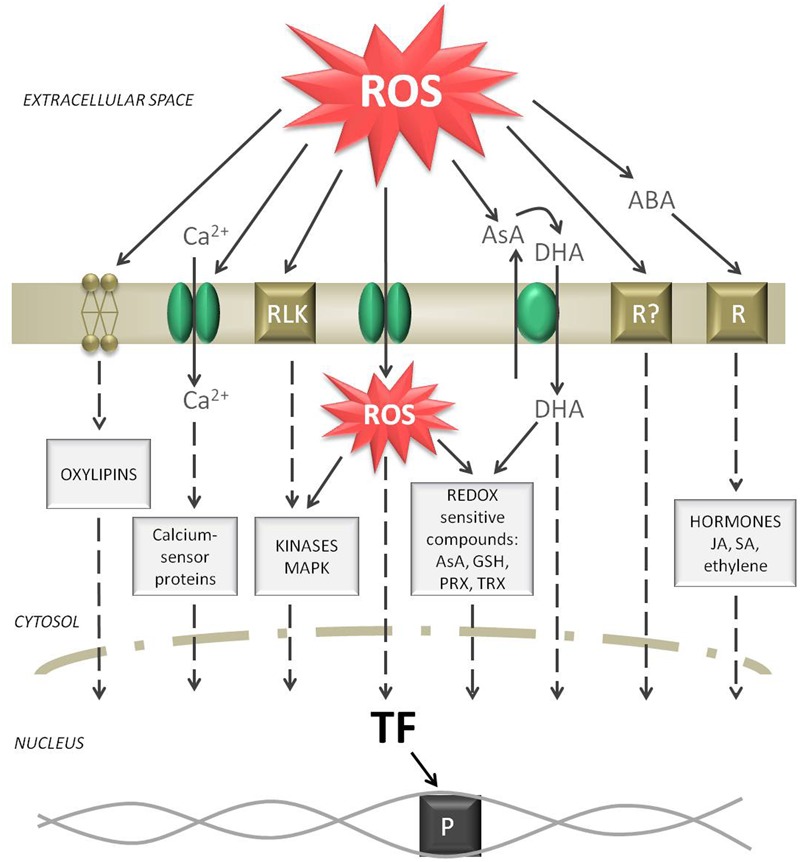
Signaling routes starting from a ROS burst in the apoplast. Elevated ROS levels can oxidize membrane lipids, activate Ca^2+^ channels, activate receptor like kinases (RLK), diffuse through aquaporins, oxidize AsA while producing DHA, activate putative ROS receptors, and induce abscisic acid (ABA) signaling. All these events form separate or common signaling cascades in the cytosol and can finally induce transcription factors (TF). As a result, the reception of a ROS burst in the apoplast can be transferred to induce polymerase (P) and start gene expression in the nucleus.

It is typically assumed that the plasma membrane serves as the first site of perception of environmental changes. Compartment-specific ROS accumulation can activate diverse signal transduction pathways (**Figure 3**). ROS signaling is not only controlled by ROS production and scavenging; the sequence of events is far more complicated. However, the signaling intermediates between stress perception in the apoplast and the physiological responses are still not fully understood (reviewed by Mittler et al., 2011; Shapiguzov et al., 2012; Baxter et al., 2014; Kangasjärvi and Kangasjärvi, 2014).

When H_2_O_2_ generation in the apoplast exceeds the relatively low scavenging capacity of this space ROS can accumulate. The first possibility is that H_2_O_2_, which is a neutral molecule, can diffuse through the plasma membrane to the cell, boosting the intracellular ROS pool and activating symplastic ROS signaling. Bienert et al. (2007) provided evidence for H_2_O_2_ diffusion through specific aquaporins. The second possibility is that the H_2_O_2_ wave can move within the apoplast from cell to cell of the same tissue (Mittler et al., 2011). For long distance transport ROS can move through the vascular system of plants. It is not surprising that the highest content of H_2_O_2_ within a leaf was detected in veins (Orozco-Cardenas and Ryan, 1999). Therefore, ROS participate in long distance communication, where the information has to be carried systematically and might be transformed into another signal to reach more distant plant organs (Baxter et al., 2014). The speed of the ROS wave was estimated to be approximately 8 cm⋅min^-1^ (Miller et al., 2009).

A major function for RBOHD of responsibility for generating fast-moving ROS signals during stressful conditions was proposed. RBOH activity is crucial for both long distance ROS signaling and the ROS burst that is transduced to the nucleus. ROS produced by RBOHs are thought to activate calcium plasma membrane hyperpolarization-activated Ca^2+^ channels (Foreman et al., 2003). Opening of these channels leads to increases in cytosolic Ca^2+^ content and causes membrane depolarization (Mittler et al., 2011). In turn, increased symplastic Ca^2+^ content can activate RBOH activity by binding the cytosolic EF-hand motif creating a feedback loop (Sagi and Fluhr, 2001). Elevated cytosolic Ca^2+^ concentrations can initiate further responses via the action of Ca^2+^-binding proteins. In plant cells these proteins can be considered calcium sensors, including calmodulins (CaMs) and CaM-like proteins (McCormack et al., 2005), calcineurin B-like (CBL) proteins (Luan, 2009), and calcium-dependent protein kinases (CDPKs or CPKs) (Harmon et al., 2000). Similar to ROS, the ubiquitous second messenger Ca^2+^ seems to be another critical step toward initiating defense signaling (Steinhorst and Kudla, 2013). The Ca^2+^ influx to the cytosol can be accompanied by other ion fluxes through dedicated channels [H^+^ pump, anion channel, stelar K+ outward rectifier (SKOR)] or permeable membranes. Hence H^+^ influx and K^+^ or Cl^-^ efflux is one of the earliest responses in ROS-mediated signaling. It was shown that K^+^ flow through SKORs is a rapid response to apoplastic H_2_O_2_ (Garcia-Mata et al., 2010). Alterations in ion conductance might be a more general signaling pathway under oxidative stress. Not only the ion balance but also the pH of the apoplast may vary since the buffering capacity in this compartment is not as efficient as that of the symplast (Grignon and Sentenac, 1991). The pH of the apoplast, which is usually between 4.5 and 5.5, may fluctuate in response to external and internal stimuli and signals (Felle, 2001). Changes in extracellular pH impacts several enzyme activities, for instance cell wall POX. Moreover, a shift in the external pH was found to be a rapid modulator of plant gene expression (Lager et al., 2010).

The major antioxidants regulate the steady-state level of ROS and therefore determine signal transduction. Since the redox state of ascorbate and glutathione pools is directly linked to ROS levels, these low-mass antioxidants might be a redox link between the apoplast and the cytosol (Sierla et al., 2013). This checkpoint might control cell cycle progression, and thereby cell growth, during stressful conditions as indicated by Reichheld et al. (1999). The ascorbate pool is not only the most important low-mass antioxidant but is a well-established element of ROS signaling (Pastori et al., 2003; Pignocchi and Foyer, 2003). Since AsA can be directly oxidized by ROS, the increased levels of DHA in the apoplast can function as signaling molecules. It was shown that the redox state of ascorbate varies throughout the cell cycle phases (Horemans et al., 2000; Pignocchi and Foyer, 2003). Apoplastic ascorbate is engaged in the regulation of the cell cycle (Kato and Esaka, 1999; Potters et al., 2000). It was proposed that increased DHA oxidation in the apoplast and elevated import to the cell can affect cell cycle progression in tobacco cell cultures (De Pinto et al., 1999; Potters et al., 2000). Furthermore, apoplastic DHA seems to inhibit cell proliferation in several plant species (Paciolla et al., 2001; Potters et al., 2004). Moreover, soluble thiol-containing low-molecular-mass compounds, mainly GSH, are important regulatory compounds in plant cells. Li et al. (2013) proposed that extracellular GSH in plants has a similar function to that of a neurotransmitter in animal cells. Moreover, GSH itself was proposed to be an activator of TFs (Tron et al., 2002). There are also several redox-sensitive proteins that operate through switching “on” and “off” depending on the cellular redox state or ROS action. All enzymes that have sulfur residues (particularly PRXs, GRXs, and TRXs) are susceptible to reversible oxidation/reduction (Spadaro et al., 2010). These redox active compounds directly modify cellular metabolism or can further execute their function via downstream signaling components.

When ROS content in the apoplast is not effectively scavenged and exceeds a certain level, it might have a toxic effect and cause tissue damage, such as protein oxidation or non-enzymatic breakdown of lipids. Oxidized PUFAs, which may be embedded in the surface of the cell as free PUFA, represent the first step in the chain reaction of lipid peroxidation. Further lipid peroxidation products from the cellular membrane, called oxylipins, can act as signaling elements (Farmer and Mueller, 2013). ROS can also oxidize extracellular peptides or domains of receptor proteins in the apoplastic space. Putative ROS receptors were speculated to exist, but to date specific apoplastic receptor proteins that can be directly modified by ROS have not been identified (Kangasjärvi and Kangasjärvi, 2014). However, there are many receptor proteins at the outer surface of the plasma membrane, including hormone receptors, innate immune receptors and others that are sensitive to ROS. For instance, animal G protein signaling is also involved in many stress-associated physiological processes in plants. The heterotrimer present in the plasma membrane after perceiving extracellular stimuli, such as apoplastic ROS accumulation, can transfer the signal to the cytosol. It was proposed that RBOHs received initial signals from G proteins working in the same signaling network. In general, the increased level of apoplastic ROS can be directly monitored by oxidizing putative receptors present in the plasma membrane that can trigger downstream signaling events. Receptor-like kinases (RLKs) in the plasma membrane can be activated by apoplastic ROS. RLKs are cysteine-rich transmembrane proteins that contain an extracellular ligand binding domain and a conserved intracellular protein kinase domain. This could be one of the mechanisms by which extracellular ROS can directly activate intracellular signaling cascades via protein phosphatases and kinases (histidine kinases) (Neill et al., 2002). Reversible protein phosphorylation is a fast signaling mechanism that can amplify the response to ROS (Baxter et al., 2014). The mitogen-activated protein kinase (MAPK) pathway is the most prominent and best studied signaling cascade. These proteins have kinase activity and are thereby interlinked. Phosphorylated (activated) MAPK interacts with and alters the phosphorylation status of target proteins, including TFs, enzymes, and other proteins, ultimately influencing gene expression; specific intermediates are still being identified. *Arabidopsis* encodes 10 MAPKKKKs, 80 MAPKKKs, 10 MAPKKs, and 23 MAPKs which form complex signaling networks (Jonak et al., 2002). Some kinases were identified as playing specific roles in ROS-mediated signaling; these include the ROS-responsive MAPKKK, MEKK1, MPK3, MPK4, and MPK6 (Colcombet and Hirt, 2008). The application of H_2_O_2_ to *Arabidopsis* protoplasts was found to activate the phosphorylation cascade involving MPK3 and MPK6 (Kovtun et al., 2000). MEKK1–MPK4 kinase activity is also activated by exogenous H_2_O_2_ treatment of *Arabidopsis* seedlings (Nakagami et al., 2006). In many conditions, proteins from the WRKY family work downstream of MAPK and can directly activate gene transcription (Eulgem and Somssich, 2007; Chen et al., 2012). A specific characteristic of plant transduction pathways is negative regulation, where a response is induced by inactivating repressor proteins. This mechanism includes protein dephosphorylation or ubiquitination, and only then can transcription proceed. Rosenfeld et al. (2002) suggested that this negative regulation provides a faster transduction system. ROS can also directly oxidize diverse proteins triggering a metabolic response. Some oxidized proteins are targeted to be degraded via the ubiquitination pathway. Other oxidized peptides were found to act directly as signaling molecules. Therefore, the abundance of diverse oxidized proteins may regulate plant responses.

Apoplastic ROS can elicit plant hormone signaling. H_2_O_2_ interaction with specific hormonal compounds, mainly salicylic acid (SA), ethylene, jasmonic acid (JA), and abscisic acid (ABA), is well established (Xia et al., 2015). All these hormones may be perceived by plasmalemma-bound hormone receptors or can further activate gene expression or MAPK signaling (Santner and Estelle, 2009). Most genes that were sensitive to apoplastic ROS were also responsive to ABA signaling (Blomster et al., 2011). ABA is localized in all cellular compartments, but it was shown with immunogold labeling that the apoplast is a major site of free ABA accumulation (Bertrand et al., 1992) or transport (Sauter and Hartung, 2000). ABA has an important role in signal transduction pathways (Wilkinson and Davies, 2002). Most reports have demonstrated that exogenous application of ABA may promote H_2_O_2_ accumulation in the apoplast, which is dependent on RBOH activity and Ca^2+^ channels (Kwak et al., 2003; Hu et al., 2005). Interaction of ABA with other hormones was also shown in the regulation of diverse physiological processes, e.g., stomatal closure. JA, SA, and ethylene are considered second messengers in oxidative signal transduction; ROS act upstream and downstream of these hormones. On one hand ethylene and SA are generally induced by oxidative bursts, on the other hand ethylene and SA signaling promote enhanced ROS production forming a self-amplifying loop (Watanabe et al., 2001; Rao et al., 2002). Experimental work showed that the application of SA can induce extracellular $O_{2}^{•–}$ production, which is probably catalyzed by cell wall POX (Kawano et al., 1998; Mori et al., 2001). Auxins can also induce $O_{2}^{•–}$ production in the apoplast, and subsequent OH⋅ release can promote elongation growth in a POX-dependent reaction (Schopfer et al., 2002). However, JA has the opposite effect than ethylene and SA, acting as an antagonist to in the regulation of different stress responses (Lorenzo and Solano, 2005). Moreover, JA was found to have the ability to reduce RBOH-induced ROS generation (Denness et al., 2011).

All of the elements described above can activate specific TFs in the nucleus. Important TF for intracellular signaling pathways that can be activated in response to ROS can belong to different families including: WRKY, NAC (NAM, ATAF1/2, and CUC2), zinc finger transcription factors (ZAT), basic-domain leucine-zipper proteins (bZIP), heat shock proteins (HSP), and others (Singh et al., 2002; Van Breusegem et al., 2008). ROS are thought to activate TFs via direct oxidation. H_2_O_2_, $O_{2}^{•–}$, and ^1^O_2_ ROS can all induce transcription of specific sets of genes in plant cells (Neill et al., 2002; Gill and Tuteja, 2010). A common TF binding site for all oxidative stress-responsive genes has never been identified, but in several ROS-induced genes specific redox-sensitive promoters have been found by Desikan et al. (2001). More specific *cis*-regulatory elements (CRE) in gene promoter regions, functioning as binding sites for TFs, have been identified in the *Arabidopsis* genome using a universal algorithm (Geisler et al., 2006). In this microarray study well-characterized motifs such as the ABA-responsive CRE (ABRE), drought responsive element (DRE), and ethylene responsive element (ERE) were analyzed upon H_2_O_2_ treatment and let to the identification of 128 putative motifs in these functional CRE, that are engaged in abiotic stress signaling.

Extracellular ROS, in particular, can control several genetic defense or developmental programs. Several approaches have been used to simulate ROS production in the apoplastic space in order to reveal the expression of specific genes. A microarray study showed that mainly cellular antioxidant enzymes, but also apoplastic proteins including RBOHs and ascorbate oxidases, were activated by H_2_O_2_ treatment, while others such as POXs and expansins were downregulated (Stanley Kim et al., 2005). Interestingly, genes that are potentially involved in signaling, including TFs of the WRKY family, were downregulated. Using mass spectrometry Wan and Liu (2008) identified different protein spots in 2DE-gel that were responsive to H_2_O_2_ treatment in rice. Upregulated proteins were mainly engaged in cell defense, redox homeostasis, signal transduction, protein synthesis and degradation, photosynthesis, and carbohydrate/energy metabolism. Rice seedling proteome changes were also analyzed in the apoplasts of roots, revealing that 45% of proteins that were responsive to H_2_O_2_ treatment were mainly involved in carbohydrate metabolism, redox homeostasis, cell defense, and signal transduction (Zhou et al., 2011). However, it should be kept in mind that exogenous treatment with H_2_O_2_ cannot be compared to *in vivo* apoplastic ROS generation.

For decades, there has been speculation as to how responses to apoplastic ROS can be so highly specific, since so many levels are engaged in signaling, and coordinated changes in gene expression and optimization of metabolic pathways are involved. This enables optimization of growth and development and survival upon environmental challenges. The ROS burst in the apoplast may be a fast on/off mechanism to induce signaling, but other signaling elements represent the specificity of signaling to different stimuli since these signaling pathways are not linear.

## Apoplastic ROS Metabolism During Environmental Stresses

Plants have developed diverse mechanisms to perceive and react to abiotic and biotic stresses (Baxter et al., 2014). In particular, an apoplastic ROS burst induced by extracellular stimuli is one of the earliest events in many plant defense responses. Environmental stresses that particularly affect the apoplastic space of plants are explored below.

### Ozone

Ozone (O_3_) exposure is very toxic to plants, and it is therefore considered one of the major air pollutants whose levels are becoming severely dangerous for crop production (Chameides et al., 1994). O_3_ has a strong oxidizing potential (+2 eV) and can directly cause injury to all cellular compounds. Ozone enters the plant leaf cell through stomata. Since this molecule is so reactive, it cannot penetrate deep into the cells; therefore, the direct site of action for O_3_ has been suggested to be the apoplast. This stress factor is often considered a model for apoplastic ROS generation (Baier et al., 2005). The mode of O_3_ action in the apoplast can be either direct, due to the strong chemical reactivity of this molecule, or indirect due to production of other ROS. The toxicity of O_3_ can be greatly enhanced by its spontaneous decomposition to ROS in aqueous environments like that of the apoplast. Ozone can act as an abiotic elicitor inducing an oxidative burst in the apoplastic space. Two hours after sunflower plants were exposed to O_3_, H_2_O_2_ deposits were detected in the apoplast by cytochemical staining (Ranieri et al., 2003). This ROS accumulation was probably connected to increased RBOH and POX activity.

In the apoplast unsaturated fatty acids in the membrane are the first to be oxidized during the ozonolysis process, therefore O_3_-induced lipid peroxidation is a major consequence. In further steps, lipid oxidation products decompose to form organic radicals, and ROS are also produced as byproducts. One of the consequences of ozone action is plasma membrane permeability and ion leakage. Other air pollutants, such as SO_2_ and NO_2_, are also dangerous to plants.

### Heavy Metals

Depending on their oxidation states, heavy metals, such as Cd, Mo, Ni, Zn, and Al, can be highly reactive and toxic for plants. When plant roots are exposed to heavy metals they are absorbed into the cells. As a first line of defense plants reduce uptake into root cells by restricting metal ions to the apoplast, binding them to the cell wall, or by inhibiting long distance transport. Therefore, the apoplast functions to accumulate heavy metals to protect intracellular metabolism and prevent the plant from suffering toxicity effects (Brune et al., 1995). Probably the most dangerous characteristic of some redox active transition metals, such as Cu^+^, Fe^2+^, and Mn^2+^ which have unpaired electrons, is their participation in the Fenton/Haber-Weiss reaction. Therefore, the most frequently documented and earliest consequence during heavy metal exposure is the overproduction of ROS in the apoplast of many plants (Pourrut et al., 2007; Zhang F. et al., 2009; Źróbek-Sokolnik et al., 2009). Increased expression of RBOHs or LOXs were frequently observed during heavy metal stress including Cd, Cu, Zn (Remans et al., 2010, 2012; Cuypers et al., 2011). On the other hand, heavy metals can inactivate important enzymes by binding to their cysteine residues or displacing cofactors. Hence, the accumulation of metals can affect activities of antioxidant enzymes, such as POXs (Kimura et al., 2014), SODs, or CATs in the apoplast, and their inactivation can be dangerous for the plants (Bhaduri and Fulekar, 2012). Moreover, heavy metals can initiate peroxidation of membrane lipids. Another dangerous feature of heavy metals that are loaded to the apoplast is that they can be immobilized in cell walls. Some of these metals may displace Ca^2+^ from the cell walls affecting their structure.

### Water Deficit

Water deficit is a common stress factor and can be observed during drought, salinity, and chilling stress which all create the problem of low water availability for plants. A water deficit initially causes loss of free water in the apoplasts of plants (Kar, 2011). This change in water content in the apoplast may impact on ROS concentrations in this space, directly affecting apROS homeostasis (Noctor et al., 2014). During water deficit plants avoid transpirational loss of water by closing stomata. It is known that stomatal movement is regulated by ion fluxes through the plasma membrane and also by hormone and ROS signaling (Munemasa et al., 2013). It was proposed that apoplastic ROS accumulation actively participates in the initiation of stomatal closure. Hence, ROS accumulation in the apoplasts of plants was observed during salinity and drought stress, which is probably connected to increased RBOH activities (Hernández et al., 2001; Evans et al., 2016).

### Temperature Stress

Changes in environmental temperatures directly affect plasma membrane stability. In response to heat stress an increased membrane fluidity can activate putative Ca^2+^ channel, which is one of the primary heat sensors in plants (Gong et al., 1998; Saidi et al., 2009). The induction of these Ca^2+^ channels leads to an increase in cytosolic calcium level. The influx of Ca^2+^ to the cytosol activates RBOH by promoting its phosphorylation (Saidi et al., 2010). It was shown that the knockout for RBOHD in *Arabidopsis* mutants impairs heat tolerance (Larkindale et al., 2005). As a consequence heat stress is accompanied by RBOH-dependent ROS generation at the plasma membrane (Königshofer et al., 2008). The ROS burst in the heat shock response can further lead to the induction among other signaling elements the activation of HSP (Kotak et al., 2007). HSPs are the most important stress proteins toward heat stress tolerance, because they act as chaperones and function in protecting proteins (Sun and Guo, 2016).

Chilling stress provides membrane destabilization and its dysfunction. Furthermore, freezing temperatures result in extracellular crystal formation affecting apoplastic metabolism. RBOH-dependent ROS formation in the apoplast was observed in response to cold stress (Zhou et al., 2012). The consequences of cold stress can be oxidative damage to lipids, protein, and other macromolecules.

### Pathogen Attack

Plant pathogens have developed diverse mechanisms to invade plant tissues. To penetrate into the cell pathogens first have to digest the cell wall, which is the main barrier. Other pathogens can penetrate plant cells through stomata. During both plant-pathogen interactions the apoplast is the first compartment that is in contact with the pathogen. In the plasmalemma plants have specific receptors (pattern recognition receptors; PRRs) that recognize pathogen elicitors – the so-called pathogen associated molecular pattern (PAMPs) (Monaghan and Zipfel, 2012). Immediately after recognition of a pathogen infection, plants activate signaling cascades and defense responses. One of the common protective mechanisms of plants is the hypersensitivity response (HR) in which local cell death occurs at the site of infection. An oxidative burst, mainly performed by RBOHs, is one of the earliest events in the HR. In 1983, Doke detected strong $O_{2}^{•–}$ generation in potato tubers prior to HR elicited by *Phytophthora infestans* and tobacco mosaic virus. Increased ROS production in response to pathogens was expected to occur due to RBOH and POX activities (Bolwell et al., 2001). A major ROS outbreak close to the pathogen may on one hand kill the pathogen directly, and on the other hand can damage the tissue of the host inhibiting spread of the pathogen.

The first action of a pathogen is to secrete diverse lytic enzymes that can further digest the cell wall. Therefore, one of the plant defense mechanisms during pathogen invasion is cross linkage of cell walls. Stiff wall polymers (lignins or callose) can protect the walls from degradation. Plant POXs are widely known to function in cross linkage of wall polymers. In the case of pathogen attack, most antioxidants do not work against the invader but are important in ROS/redox signaling cascades. In the fight against the pathogen plants can very rapidly produce specific toxins, hydrolytic enzymes, pathogenesis-related proteins, and other substances.

Interestingly most environmental stresses that are not directly connected to the apoplast, such as strong light, ion imbalance, and wounding, can also affect apoplastic ROS metabolism. Increased ROS levels have been frequently observed during exposure to all these stresses. Microarray data showed that almost all stresses can induce RBOH expression in plants (Suzuki et al., 2012). Intracellular signaling can probably connect cellular stresses to trigger apoplastic ROS production. It was proposed that ROS production in organelles can trigger RBOH-dependent systemic signaling. For instance, it was observed that the impairment of mitochondrial complex I in the *frostbite* mutant, which led to oxidative stress in mitochondria, also affected apoplastic ROS metabolism (Podgórska et al., 2015). Mitochondrial or chloroplastic ROS generation during metabolic fluctuations can induce ROS production in extracellular spaces. The interplay between the apoplast and organelles needs further research.

## Conclusion

Further research is needed to understand apROS metabolism in detail. In this review, we have tried to emphasize how important this specific aspect is for plant metabolism. ApROS are produced and detoxified in enzymatic and non-enzymatic reaction depending on numerous factors, including apoplast pH, metal ion concentration, or availability of metabolites in the extracellular space. ApROS allow rearrangements of the cell wall, and in consequence the growth of plants. Products of apROS metabolism regulate the rate of cells division. The apoplast is the first place to receive signals coming from the outside, and apROS are involved in both local defense and the signal transmission into the cell and other plant organs. The results of experiments performed using plants with altered expression of apROS producing enzymes (**Table 2**) clearly indicate the importance of apROS in plant development and plant–pathogen interaction. It can be assumed that genetic manipulation of the expression of enzymes involved in apROS metabolism may allow the breeding of crop plants more resistant to pathogens, with greater biomass, or a desired lignin profile. However, it should be noted that apROS metabolism is a very complex multi-piece puzzle. Changing one of its elements may not lead to the desired effect because of (i) the multitude of enzyme isoforms (e.g., 10 RBOHs and over 70 isoforms of POX exist in *Arabidopsis*) that can take over its functions, (ii) individual compound cross-regulation (action of RBOHs can be regulated by POX activity), and (iii) the opposing reactions in which they are involved (e.g., POXs are ROS-producing and ROS-detoxifying enzymes; polyamines are antioxidants but also substrates for ROS- producing enzymes).

**Table 2:** Impact of changes in apROS-producing enzyme expression on development and metabolism of *Arabidopsis* plants (examples).

Enzyme	Changes in gene expression	Developmental/metabolic effect	Reference
**NADPH Oxidases**			
RBOHC	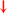 At5g51060	Stunted roots, short root hairs, defect in setting up the tip-high calcium gradient in root hairs	Foreman et al., 2003; Monshausen et al., 2007; Takeda et al., 2008
RBOHD	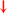 At5g47910	Smaller rosette size; enhanced accumulation coumarin and scopoletin in response to pathogen treatment; decreased accumulation of callose in response to elicitor; suppression of jasmonic acid-responsive genes; inability to mediate rapid systemic response; higher sensitivity aphid infection	Galletti et al., 2008; Miller et al., 2009; Maruta et al., 2011; Chaouch et al., 2012
RBOHF	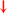 At1g64060	Delayed accumulation of salicylic acid in response to pathogen treatment; decreased APX1 transcript level suppression of jasmonic acid-responsive genes	Maruta et al., 2011; Chaouch et al., 2012
RBOHH/ RBOHJ	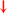 At5g60010/ 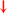 At3g45810	Reduced fertility; pollen tube collapse	Lassig et al., 2014
**Peroxidases**			
POX2 or POX25	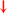 At1g05250 or 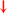 At2g41480	Lower dry-weight of the main stem; decreased lignin content and altered lignin structure	Shigeto et al., 2013, 2015
POX34	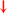 At3g49120	Reduced callose deposition in response to microbe-associated molecular patterns; diminished expression of flagellin-responsive genes	Daudi et al., 2012
POX37	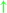 At4g08770	Dwarfism; smaller leaf surface; delay on growth of the flower stems; reduction in root length and secondary root development; increase in phenolic compounds of cell wall	Pedreira et al., 2011
POX53	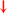 At5g06720	Longer hypocotyl; increased susceptibility to nematode; three-carpel silique phenotype	Jin et al., 2011
POX57	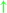 At5g17820	Dwarfism; increased callose deposit in response to bacterial elicitors; increased resistance to nectrotrophic pathogens and higher sensitivity to hemibiotroph pathogens; impaired cutin biosynthesis	Lu et al., 2014; Survila et al., 2016
POX 71	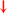 At5g64120	Enhanced biomass; longer hypocotyls; increased cell area	Raggi et al., 2015
	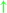 At5g64120	Reduction of biomass; reduction of cell size	
**Amine Oxiades**			
AtCuAOβ (CuAO1)	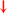 At1g62810	Impaired NO production; lower sensitivity to exogenous ABA; lower expression of stress-responsive genes in response to ABA treatment	Wimalasekera et al., 2011

## Author Contributions

AP, MB, and BS wrote the manuscript. All authors read and approved the manuscript.

## Conflict of Interest Statement

The authors declare that the research was conducted in the absence of any commercial or financial relationships that could be construed as a potential conflict of interest.
